# A Case of Painful Os Styloideum in the Midfoot (Tarsal Boss)

**DOI:** 10.7759/cureus.23182

**Published:** 2022-03-15

**Authors:** Kristina Dolenc, Mitja Rupreht

**Affiliations:** 1 Radiology, University Medical Centre Maribor, Maribor, SVN

**Keywords:** midfoot pain, bone marrow edema, magnetic resonance imaging, tarsal boss, tarsal os styloideum

## Abstract

A case of symptomatic bone fragment without trauma in the midfoot at the dorsal aspect between first and second tarsometatarsal joints is presented. Such fragment is a common finding in the wrist, referred to in the literature as an os styloideum or carpal boss; however, it has not yet been described in a similar location in the midfoot.

## Introduction

Lumps on the dorsal side of the foot and ankle represent common patient complaints, which can manifest as pain, limited motion, or even concerns about the possibility of a tumor. Irritation from shoes or straps, unintentional bumping, and various work and sports activities can additionally result in significant discomfort. Such prominences can represent accessory bones, malunited fragments of previous bone fracture, osteophytes, malformed or misaligned bones, soft tissue masses, or fluid collections. In particular, small anatomical variants in the foot and ankle area can be overlooked at imaging [1]. Hereby, a rare case of tarsal os styloideum resulting in a painful lump at the dorsal foot, identified through MRI examination, is presented.

## Case presentation

A 24-year-old male patient presented to the physical medicine specialist with pain in the right midfoot between the first and second metatarsals proximally, which lasted for a year and a half. He also experienced a tingling sensation in his big toe. The pain worsened while riding a bicycle, running, or walking. He could not recall any injury. During the examination, the patient expressed pain to palpation in the proximal web space between the bases of the first and second metatarsals. The transverse arch of the right foot appeared flattened. Ultrasound examination showed no signs of neurinoma or injury of the Lisfranc complex.

**Figure 1:**
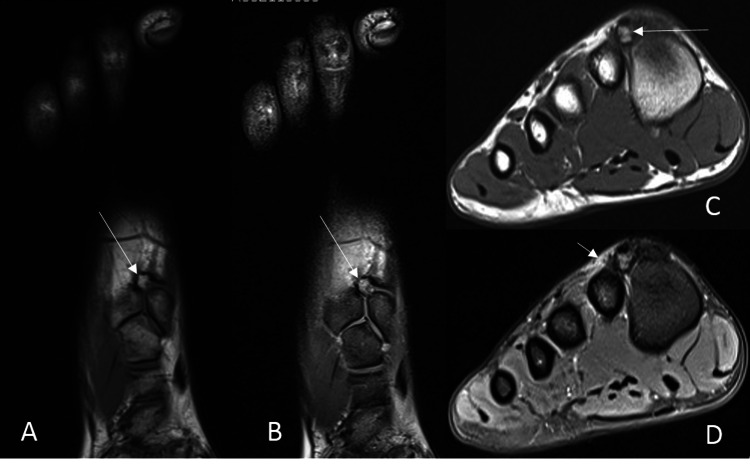
Tarsal os styloideum. MRI of the right foot. Coronal proton-density (PD) images without (A) and with (B) fat suppression. Transverse T1 (C) and PD fat-suppressed (PDFS) (D) images demonstrate bone fragments at the dorsal aspect between the first and second tarsometatarsal joints (long arrows). On sagittal images (not shown), the fragment was not visible owing to the slice thickness limitation. Note the high signal intensity of the fragment on PDFS images (B, D) indicating bone marrow edema of the fragment. Also note the increased PDFS signal (short arrow in D) indicating possible soft-tissue edema, although it could also be attributed to the incomplete fat suppression, which is possible in this location.

MRI examination demonstrated a small bone fragment at the dorsal aspect between the first and second metatarsophalangeal joint with a bone marrow edema signal as well as some edema signal of the surrounding soft tissues on the fluid sensitive sequences (Figure 1). The marrow signal of adjacent bones had normal intensity. The signal intensity of both toe extensor tendons running near the fragment was preserved. The patient was referred to the orthopedic surgeon for further workup.

## Discussion

**Figure 2:**
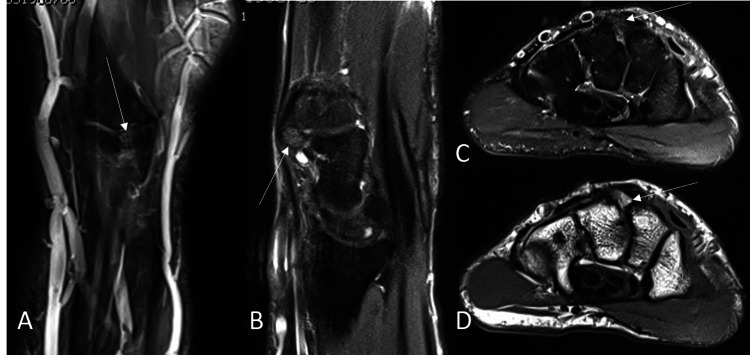
Typical carpal os styloideum. A 39-year-old male with dorsal left wrist pain. Proton-density fat-saturated (PDFS) MRI in coronal (A), sagittal (B), and transverse (C) plane, as well as T1-weighted transverse image (D), demonstrate a small bony fragment at the dorsal aspect of the wrist between the second and third carpometacarpal joint (arrows). Note the high signal intensity of the fragment on PDFS images indicating bone marrow edema of the fragment.

The carpal boss (os styloideum) in the wrist is a common finding. It was first described by Fiolle and Ailland in 1932 as a fixed dorsal protuberance at the base of the second and third metacarpals [1,2]. It represents an accessory ossicle located dorsally between the trapezoid, capitate, and the second and third metacarpals (Figure 2). Cadaver studies reported that the prevalence of carpal boss is up to 19%. It is mostly asymptomatic. Around 1% of affected people report pain in the dorsal wrist while performing strong mechanical or repetitive work. Most often, it affects the dominant hand and both hands in 10-20% of cases. It is mostly seen in young adults (20-50 years) with no gender preference [3]. The fragment can irritate the surrounding soft tissues, including extensor tendons, with possible bursa formation over the bony fragment. It can also interfere with normal biomechanics of the surrounding joints and lead to degenerative osteoarthritis [4]. The cause of the symptoms could also be slipping of the extensor tendon over it. Patients may report a history of direct trauma causing an injury, and in other cases, symptoms may be secondary to sports like golf or racket sports [2].

To the best of our efforts, we could not identify a single publication describing a bone fragment in the midfoot analog to the carpus. While the search in PubMed with keywords “carpal boss” at the time of preparing the manuscript yielded 64 results and with “os styloideum” yielded 26 results with a common overlap with the previous 64 results, the search with the added word “tarsal” yielded only two results, respectively, both describing dorsal osteophytes or bony spurs rather than a true bone fragment [5,6]. Such dorsal protuberances of the foot can cause pain, blisters, or subcutaneous pressure lesions as a result of footwear compression. Other findings include ganglion cysts, adventitious bursitis, or extensor tendonitis [5]. Treatment of such lesions can be conservative, which includes loosening of the shoelaces, soft padding between the shoe and the tarsal boss, and stiff-soled shoe. These actions decrease the stress to the arthritic midfoot joints. If the symptoms do not improve with conservative measures, surgery is indicated. The operative treatment of choice is excision of the dorsal boss with or without arthrodesis of the underlying joint. Fusion of the underlying joints is indicated in cases with significant arthritic changes that cause pain while walking barefoot [5].

In the presented case, the bony fragment could represent an old abruption; however, any injury has been categorically denied by the patient. Additionally, no defects of the neighboring bones were identified on MRI.

The patient also complained of unusual sensations in the first toe. This could represent a possible entrapment of the cutaneous nerve or deep peroneal nerve [5,7]. A positive Tinel sign is used as a diagnostic tool for chronic nerve compression at the dorsal sensory territory of the medial cuneiform, with sending sensation to the first and second digits. The branching pattern of the deep peroneal nerve seems to vary, as in some cases, the nerve does not provide innervation to the first web branches. In other cases, it may provide innervation to the inner side of the big toe as well as to the second and third web branches [7]. The electromyography examination was not performed, so this could not be verified.

## Conclusions

To the best of our knowledge, this is a first documented case of a bone fragment in the midfoot dorsal next to first and second tarsometatarsal joints, a so-called tarsal boss, with the edema of the bone marrow and surrounding soft tissues as well as clinical symptoms of deep peroneal nerve compression. While such bone fragments are frequently found in the wrist, where they are referred to as the carpal boss, we could not find any publication on analog pathology in the midfoot. The presented case should alert radiologists as well as clinicians of another possible curable cause of the dorsal midfoot pain.
